# Association between long-term usage of acetylcholinesterase inhibitors and lung cancer in the elderly: a nationwide cohort study

**DOI:** 10.1038/s41598-022-06377-3

**Published:** 2022-03-03

**Authors:** Chien-Ting Liu, Chuan-Chi Yang, Wu-Chien Chien, Chi-Hsiang Chung, Chien-Sung Tsai, Yi-Ting Tsai, Chih-Yuan Lin, Yi-Chang Lin, Yi-Shi Chen, Nian-Sheng Tzeng

**Affiliations:** 1 Division of Cardiovascular Surgery, Department of Surgery, Tri-Service General Hospital, School of Medicine, National Defense Medical Center, Taipei, Taiwan; 2 Department of Psychiatry, Taoyuan Armed Forced General Hospital, School of Medicine, National Defense Medical Center, Taipei, Taiwan; 3 Department of Medical Research, Tri-Service General Hospital, National Defense Medical Center, 7115R, No. 325, Section 2, Cheng-Kung Road, Neihu District, Taipei, 11490 Taiwan; 4 Graduate Institute of Life Sciences, National Defense Medical Center, Taipei, Taiwan; 5 School of Public Health, National Defense Medical Center, Taipei, Taiwan; 6 Taiwanese Injury Prevention and Safety Promotion Association, Taipei, Taiwan; 7 Division of Thoracic Surgery, Department of Surgery, Tri-Service General Hospital, Keelung Branch, National Defense Medical Center, Keelung, Taiwan; 8 Department of Psychiatry, School of Medicine, Tri-Service General Hospital, National Defense Medical Center, 325, Section 2, Cheng-Gung Road, Nei-Hu District, Taipei, Taiwan; 9 Student Counseling Center, National Defense Medical Center, Taipei, Taiwan

**Keywords:** Neurology, Oncology, Risk factors

## Abstract

This retrospective cohort study aimed to evaluate the association between acetylcholinesterase inhibitors (AChEI) usage and the risk of lung cancer. Data from 116,106 new users of AChEI and 348,318, at a ratio of 1:3, matched by age, sex, and index-year, between 2000 and 2015 controls were obtained from the Taiwan Longitudinal Health Insurance Database in this cohort study. The Cox regression model was used to compare the risk of lung cancer. The adjusted hazard ratio (aHR) of lung cancer for AChEI users was 1.198 (95% confidence interval [CI] = 0.765–1.774, *p* = 0.167). However, the adjusted HR for patients aged ≥ 65 was adjusted to HR: 1.498 (95% CI = 1.124–1.798, *p* < 0.001), in contrast to the comparison groups. In addition, patients with comorbidities such as pneumonia, bronchiectasis, pneumoconiosis, pulmonary alveolar pneumonopathy, hypertension, stroke, coronary artery disease, diabetes mellitus, chronic kidney disease, depression, anxiety, smoking-related diseases, dementia, and seeking medical help from medical centers and regional hospitals, were associated with a higher risk in lung cancer. Furthermore, longer-term usage of rivastigmine (366–730 days, ≥ 731 days) and galantamine (≥ 731 days) was associated with the risk of lung cancer. AChEI increased the risk of lung cancer in the older aged patients, several comorbidities, and a longer-term usage of rivastigmine and galantamine. Therefore, physicians should estimate the risks and benefits of AChEI usage and avoid prescribing antidepressants concurrently.

## Introduction

Dementia could very well be a heavy burden for the patients and their caregivers, community, and society^1–4^, and the most common type is Alzheimer dementia (AD). Acetylcholinesterase inhibitors (AChEI), such as donepezil, rivastigmine, and galantamine, are efficacious and safe for the treatment of mild to moderate AD^5,6^, and donepezil is also effective in treating moderate-severe to severe AD^7^. In addition, previous studies have shown that AChEI usage was associated with a decreased risk of injury^8^, mortality^9^, myocardial infarction^10^, and ischemic stroke^11^.

Lung cancer is one of the leading causes of death worldwide including Taiwan. Despite all the innovations in radiology imaging diagnostic testing, surgical techniques, chemotherapy treatments, the 5-year survival rates for the lung cancer patients were just 6–18%^12^. Lacking specific biomarkers and tools for early detection, most of the patients were diagnosed at advance stages. The etiology and pathogenesis of the lung cancer are very complex. Cigarette smoking bears a strong association with lung carcinogenesis. Other contributing risk factors include the occupational or environmental exposure to the crystalline silica dust, asbestos, arsenic, residential radon, polycyclic aromatic hydrocarbons, passive smoking, and ambient air pollution. Prompt investigations for risk factors from other chemicals of lung cancer are now ongoing for improving the patients’ diagnosis and treatment survival rates^13^. However, several studies have found that the neurotransmitter acetylcholine (ACh) acts as an autocrine growth factor for human lung cancer^14^ and acetylcholinesterase (AChE) is also a promising tumor suppressor^15^. The inhibition of AChEI could increase the levels of Ach, and this finding raised a possibility of the association between AChEI and lung cancer.

Thus, there is no study, as yet, for the relationship between AChEI and the risk of lung cancer. Furthermore, no large, population-based study has comprehensively analyzed and evaluated the potential risk of lung cancer according to the age of the patients and the duration of AChEI usage. To this end, we conducted a population-based cohort study so as to determine the risk of lung cancer over 15 years.

## Results

### Baseline and end-point characteristics of the study population

Table 1 depicts the baseline characteristics of the study population. There were 116,106 subjects in the AChEI usage group and 348,318 in the non-AChEI controls, with a similar distribution of sex and age. The AChEI usage group tended to be higher in all the comorbidities, with monthly insured premiums of 18,000–34,999 and ≥ 35,000 New Taiwan Dollars (NT$), married, and education levels ≥ 12 years, geographical area of residence in the North, East, and outlying islands of Taiwan, urbanization levels 1 and 4, and levels of medical care in the medical center. Characteristics of the study at the endpoint are as illustrated in Table S2.

**Table 1:** Characteristics of study at the baseline.

AChEI	With		Without		*P*
Variables	n	%	n	%	
Total	116,106	25.00	348,318	75.00	
**Gender**					0.999
Male	59,052	50.86	177,156	50.86	
Female	57,054	49.14	171,162	49.14	
Age (years)	60.21 ± 18.25		60.31 ± 19.94		0.131
**Age groups (years)**					0.999
50–64	58,712	50.57	176,136	50.57	
≥ 65	57,394	49.43	172,182	49.43	
**Monthly insurance premiums**					0.020
< 18,000	92,246	79.45	278,012	79.82	
18,000–34,999	13,459	11.59	39,454	11.33	
≥ 35,000	10,401	8.96	30,852	8.86	
**Marital status**					< 0.001
Without	50,982	43.91	158,617	45.54	
With	65,124	56.09	189,701	54.46	
**Education levels (years)**					< 0.001
< 12	60,986	52.53	191,336	54.93	
≥ 12	55,120	47.47	156,982	45.07	
Pneumonia	8715	7.51	14,541	4.17	< 0.001
Bronchiectasis	9011	7.76	19,701	5.66	< 0.001
Pneumoconiosis	8875	7.64	16,701	4.79	< 0.001
PAP	1597	1.38	1714	0.49	< 0.001
COPD	10,875	9.37	22,472	6.45	< 0.001
Asthma	5556	4.79	15,701	4.51	< 0.001
Hypertension	22,106	19.04	64,013	18.38	< 0.001
Stroke	14,512	12.50	34,012	9.76	< 0.001
Coronary artery disease	17,541	15.11	33,451	9.60	< 0.001
Diabetes mellitus	23,895	20.58	69,123	19.84	< 0.001
Chronic kidney disease	15,872	13.67	44,012	12.64	< 0.001
Osteoporosis	3340	2.88	6124	1.76	< 0.001
Depression	6701	5.77	3978	1.14	< 0.001
Anxiety	5512	4.75	2811	0.81	< 0.001
Hyperlipidemia	5101	4.39	11,022	3.16	< 0.001
Smoking-related diseases	4841	4.17	12,901	3.70	< 0.001
Dementia	29,785	25.65	66,971	19.23	< 0.001
CCI_R	1.02 ± 1.85		0.84 ± 1.72		< 0.001
**Location**					< 0.001
Northern Taiwan	44,123	38.00	131,010	37.61	
Middle Taiwan	28,452	24.51	91,225	26.19	
Southern Taiwan	32,013	27.57	98,720	28.34	
Eastern Taiwan	10,712	9.23	25,451	7.31	
Outlying islands	806	0.69	1912	0.55	
**Urbanization level**					< 0.001
1 (the highest)	40,112	34.55	118,706	34.08	
2	45,124	38.86	139,126	39.94	
3	10,245	8.82	34,529	9.91	
4 (the lowest)	20,625	17.76	55,957	16.06	
**Level of care**					< 0.001
Medical center	44,802	38.59	105,601	30.32	
Regional hospital	42,121	36.28	128,701	36.95	
Local hospital	29,183	25.13	114,016	32.73	

*P*: Chi-square/Fisher exact test on category variables and *t* test on continue variables.

*AChEI* acetylcholinesterase inhibitors, *CCI_R* Charlson Comorbidity Index, dementia removed, *COPD* chronic obstructive pulmonary disease, *NT$* New Taiwan Dollars, *PAP* pulmonary alveolar pneumonopathy.

### The association between AChEI and lung cancer

**Figure 1:**
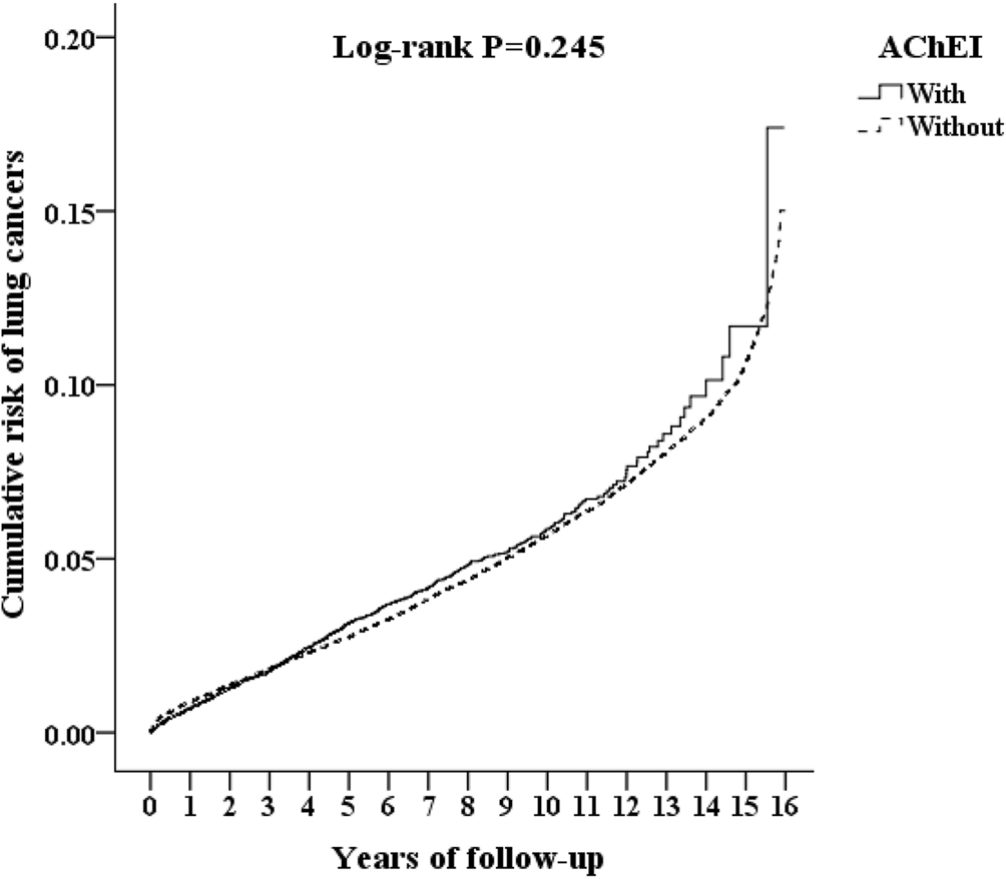
Kaplan–Meier for cumulative risk of lung cancers aged 50 and over stratified by AChEI with log-rank test.

Of the AChEI usage group, 4713 (371.04 per 100,000 person-years) suffered lung cancer when compared to 14,071 (362.52 per 100,000 person-years) in the control group, after the 15-year follow-up. Figure 1 shows the Kaplan–Meier analysis was used for the cumulative incidence of lung cancer in the AChEI users and non-user controls (log-rank test, p = 0.245).

Table 2 discloses that the Cox regression model revealed that the adjusted HR for dementia was 1.198 (95% CI = 0.765–1.774, *p* = 0.167), when compared with the controls, after adjusting for age, sex, comorbidities, CCI scores, and all the covariates. However, the adjusted HR for patients aged ≥ 65 was 1.498 (95% CI = 1.124–1.798, *p* < 0.001), in contrast to the comparison groups. The adjusted HR’s for patients with comorbidities such as pneumonia, bronchiectasis, pneumoconiosis, pulmonary alveolar pneumonopathy, hypertension, stroke, coronary artery disease, diabetes mellitus, chronic kidney disease, depression, anxiety, smoking-related diseases, dementia, and seeking medical help from medical centers and regional hospitals, were associated with a higher risk in lung cancer.

**Table 2:** Factors of lung cancers by using Cox regression model.

Variables	Crude HR	95% CI	95% CI	*P*	Adjusted HR	95% CI	95% CI	*P*
AChEI (reference: without)	1.240	0.792	1.798	0.198	1.198	0.765	1.774	0.167
Age ≥ 65 (reference: age of 50–64)	1.524	1.165	1.823	< 0.001	1.498	1.124	1.798	< 0.001
Pneumonia (reference: without)	2.501	2.134	2.801	< 0.001	2.495	2.101	2.786	< 0.001
Bronchiectasis (reference: without)	1.776	1.254	2.148	< 0.001	1.754	1.241	2.131	< 0.001
Pneumoconiosis (reference: without)	2.345	1.955	2.794	< 0.001	2.284	1.925	2.765	< 0.001
PAP (reference: without)	1.731	1.213	2.124	< 0.001	1.724	1.201	2.111	< 0.001
Hypertension (reference: without)	1.848	1.364	2.124	< 0.001	1.842	1.345	2.097	< 0.001
Stroke (reference: without)	1.767	1.303	2.048	< 0.001	1.752	1.289	2.035	< 0.001
Coronary artery disease (reference: without)	1.772	1.296	2.010	< 0.001	1.722	1.264	1.999	< 0.001
Diabetes mellitus (reference: without)	1.598	1.134	1.995	< 0.001	1.579	1.124	1.986	< 0.001
Chronic kidney disease (reference: without)	2.341	1.598	2.685	< 0.001	2.297	1.583	2.674	< 0.001
Depression (reference: without)	1.682	1.168	1.989	< 0.001	1.591	1.154	1.984	< 0.001
Anxiety (reference: without)	1.703	1.203	2.024	< 0.001	1.687	1.197	2.015	< 0.001
Smoking-related diseases (reference: without)	1.099	1.037	1.134	0.017	1.095	1.033	1.129	0.014
Dementia (reference: without)	1.089	1.026	1.129	0.024	1.087	1.024	1.124	0.026
CCI_R	1.127	1.103	1.149	< 0.001	1.124	1.101	1.145	< 0.001
Medical center (reference: local hospital)	1.602	1.131	2.005	< 0.001	1.598	1.128	1.986	< 0.001
Regional hospital (reference: local hospital)	1.467	1.108	1.996	< 0.001	1.451	1.105	1.973	< 0.001

*HR* hazard ratio, *CI* confidence interval, *Adjusted HR* Adjusted variables listed in the table 1, *AChEI* acetylcholinesterase inhibitors, *CCI_R* Charlson Comorbidity Index, dementia removed, *PAP* pulmonary alveolar pneumonopathy.

### Longer term of usage and comorbidities of AChEI and the risk of lung cancer

Table 3 shows that longer-term usage of rivastigmine (366–730 days, ≥ 731 days) and galantamine (≥ 731 days) was associated with the risk of lung cancer. Table S3 depicts that the AChEI usage patients with pneumonia and pneumoconiosis were associated with a higher risk of lung cancer in comparison with the controls without the usage of AChEI.

**Table 3:** Factors of lung cancers among AChEI subgroups by using Cox regression and Fine and Gray's competing risk model.

AChEI subgroups	Rate (per 10^5^ PYs)	Adjusted HR	95% CI	95% CI	*P*
Without AChEI	362.52	Reference			
**With AChEI**	371.04	1.198	0.765	1.774	0.167
With Donepezil	367.91	1.188	0.759	1.759	0.189
With Donepezil, 1–30 days	363.38	1.173	0.749	1.737	0.204
With Donepezil, 31–365 days	369.39	1.193	0.762	1.766	0.187
With Donepezil, 366–730 days	365.10	1.179	0.756	1.747	0.201
With Donepezil, ≥ 731 days	372.94	1.204	0.769	1.783	0.158
With Rivastigmine	369.48	1.194	0.862	1.685	0.225
With Rivastigmine, 1–30 days	362.63	1.173	0.743	1.642	0.313
With Rivastigmine, 31–365 days	364.59	1.178	0.751	1.659	0.286
With Rivastigmine, 366–730 days	373.47	1.245	1.041	1.884	0.003
With Rivastigmine, ≥ 731 days	375.29	1.297	1.043	1.889	0.001
With Galantamine	367.70	1.187	0.905	1.825	0.165
With Galantamine, 1–30 days	363.11	1.172	0.849	1.734	0.297
With Galantamine, 31–365 days	365.14	1.179	0.853	1.749	0.183
With Galantamine, 366–730 days	366.48	1.183	0.884	1.755	0.145
With Galantamine, ≥ 731 days	375.01	1.211	1.070	1.978	< 0.001

*PYs* person-years, *Adjusted HR* adjusted hazard ratio: adjusted for the variables listed in Table 3, *CI* confidence interval.

## Discussion

After adjusting for the covariates, the overall adjusted HR was 1.198 (95% CI = 0.765–1.774, *p* = 0.167), when compared with the controls. The Kaplan–Meier analysis revealed that the 15-year cumulative incidence rate between the AChEI-cohort and the controls was not significant (p = 0.245). Nonetheless, the adjusted HR for patients aged ≥ 65 was 1.498 (95% CI = 1.124–1.798, *p* < 0.001), in contrast to the comparison groups. In other words, the patients in the AChEI-cohort aged ≥ 65 had a nearly 1.5-fold increased risk of the development of lung cancer.

In addition, the patients with comorbidities, such as pneumonia, bronchiectasis, pneumoconiosis, pulmonary alveolar pneumonopathy, hypertension, stroke, coronary artery disease, diabetes mellitus, chronic kidney disease, depression, anxiety, smoking-related diseases, dementia, and seeking medical help from medical centers and regional hospitals, were associated with a higher risk of lung cancer. To the best of our knowledge, this is the first study on the topic of the association between the usage of ACEI and the risk of lung cancer.

Several reports have shown that AChEI was associated with a higher risk of bradycardia^16^, syncope^17^, and other adverse cardiovascular events^18,19^. There are no studies about the potential disadvantageous effects of AChEI except the cardiovascular events. The present study has pointed out that the future pharmacological and clinical studies are important when referring to the potential effects of carcinogenesis of the AChEI.

One study has shown that a chemical compound, eserine, and an acetylcholinesterase inhibitor, was capable of inducing carcinogenesis in the epithelium of rat mammary glands^20^. Another study reported that the Ach could act as an autocrine growth factor for human lung cancer cells^14^, the association between AChEI, which could increase the level of Ach, and the risk of lung cancer. Another study has found that it is also a promising tumor suppressor, therefore, the inhibition of the AChE would not be beneficial for the suppression of carcinogenesis^15^. The degradation of the acetylcholinesterase and butyrylcholinesterase would lead to the consequent release of acetylcholine that binds back to the nicotinic and muscarinic receptors, could accelerate their proliferation, migration, and invasion of the lung cancer cells^15^, in both the carcinogenesis and progression of lung cancer. In the present study, it would take a longer duration of rivastigmine and galantamine usage for the development of the risk, therefore, we speculate that the interaction between ACEI and the aging process might well play an important role in the pathogenesis of lung cancer.

In the present study, the association between long-term usage of rivastigmine and galantamine, but not donepezil, has been found. The underlying reasons for this difference are yet to be clarified. Not only the degradation of acetylcholinesterase, but also the butyrylcholinesterase, and the consequent release of acetylcholine that binds back to the nicotinic and muscarinic receptors could accelerate their proliferation, migration, and invasion of the lung cancer cells^15^. In addition, previous studies have shown that the decreased circulating butyrylcholinesterase predicts a shorter survival for patients with pancreatic cancer^21^, and non-muscle-invasive bladder cancer^22^. Therefore, for rivastigmine, the dual inhibition of acetylcholinesterase and butyrylcholinesterase might play an important role for the increased risk of lung cancer, especially in the long-term usage. On the other hand, although galantamine is a selective AChEI^23^, some other medications, such as escitalopram, could result in the synergistic inhibition on the butyrylcholinesterase^24^. We speculate that in the long-term usage of galantamine, the concurrent usage of other medications might be a reason for the additional inhibition on butyrylcholinesterase and the subsequent increased risk of lung cancer.

### Limitations

First, even though the NHIRD have recorded inpatient care, ambulatory care, dental care, and prescription drugs availed by the insured and their date of birth. However, pursuant to the Personal Information Protection Act, individual identifiers are encrypted before being released for research. Therefore, information such as the severity, laboratory parameters, neurological symptom severity, electrophysiological testing, or rehabilitation potential could not be assessed in the present study for dementia due to the lack of such data in the NHIRD. Besides, we could not include data on the psychosocial, environmental, and genetic factors in our analyses due to the same reason. Second, the NHIRD does not contain the data of initial chest imaging or detailed pulmonary evaluation. Third, the information for the smoking and the pack-years of cigarettes was not included in Taiwan’s NHIRD. However, in this dataset, smoking-related diseases, such as tobacco usage disorder (ICD-9-CM code: 305.1), interstitial emphysema (ICD-9-CM code: 518.1), pulmonary eosinophilia (ICD-9-CM code: 518.3–518.4), nonspecific abnormal results of pulmonary function study (ICD-9-CM code: 794.2), and personal history of tobacco usage (ICD-9-CM code: V15.82), were included, as listed in Table S1. Therefore, the role of health disadvantages for smoking has been analyzed in the present study. However, despite these limitations, our derived data are highly likely to be valid and representative due to the NHIRD containing data covering all hospitals within Taiwan and over 99% of the population for the relevant 15-year period.

## Methods

### Data sources

Taiwan’s National Health Insurance (NHI) Program was launched in 1995, and had contracts with 97% of the medical providers and enrolled more than 99% of the 23 million population, as of June, 2009^25^. The details of the program have been documented in previous studies^26–34^. We used the Longitudinal Health Insurance Database (LHID), a subset of two million randomly sampled patients from the NHIRD, during a 15-year period (2000–2015) in this study.

### Ethics approval

This study was conducted according to the Code of Ethics of the World Medical Association (Declaration of Helsinki). This study was approved by the Institutional Review Board (IRB) of the Tri-Service General Hospital (TSGH). The TSGH IRB waived the need of individual consents since all the identification data were encrypted in the NHIRD (IRB No. B-110-30).

### Study design and participants

The International Classification of Diseases, 9th Revision, Clinical Modification (ICD-9-CM)^35^ diagnostic codes were used in the NHIRD. Each diagnosis of dementia was made by a board-certified psychiatrist or neurologist according to the Diagnostic and Statistical Manual of Mental Disorders, 4th Edition (DSM-IV) and its Text-revised edition (DSM-IV-TR)^36,37^. Other causes of dementia must likewise be excluded: patients with cerebral vascular disease history were excluded. Patients with old vascular insults, hydrocephalus, brain tumor, or any other potential cause of dementia other than AD noted in these neuro-images were excluded. The Mini-Mental Status Examination (MMSE) score must be between 10 and 26 and clinical dementia rating (CDR) grade either 1 or 2. The requested blood tests include the venereal disease research laboratory, thyroid function, complete blood count, fasting sugar, glutamic–oxaloacetic transaminase, glutamic–pyruvic transaminase, blood urea nitrogen, creatinine, serum B12 and folic acid levels. Aside from the clinical presentation, cognition tests and blood tests, all of the patients must have neuro-image studies, with either a brain computerized tomogram or a magnetic resonance image. The diagnostic work-up must be performed and confirmed by a certificated neurologist or psychiatrist. The AChEI treatment for AD is covered by the NHI program. According to the NHI regulations, the AChEI medications are exclusively used in AD patients^38^.

The records of ambulatory care visits and inpatient claims by the NHI Administration are randomly reviewed, to verify the accuracy of the diagnoses^39^. Therefore, using the NHIRD is considered as being suitable to study the association between HMCAA and the risk of developing dementia. The records of the AChEI were also retrieved from the NHIRD. We also calculated the estimated cumulative dosage of AChEI for each subject using the defined daily dose (DDD) that were obtained from the WHO Collaborating Centre for Drug Statistics Methodology (https://www.whocc.no/), and the duration of the usage of AChEI was calculated by dividing the cumulative doses by the DDD of the AChEI. A total of 116,106 new users of AChEI were enrolled, along with 348,318 controls without the usage of AChEI, at a ratio of 1:3, matched by age, sex, and index-year, between 2000 and 2015, from a two million LHID, a subset of Taiwan’s National Health Research Institutes Database, in this retrospective cohort study (Fig. S1).

### Covariates

Covariates included sex, age (50–64, ≥ 65), geographical area of residence (northern, central, southern, and eastern Taiwan), urbanization level (levels 1 to 4, as described below), monthly insured premiums (in New Taiwan dollars (NT$): < 18,000, 18,000–34,999, ≥ 35,000), and levels of medical care (medical center, regional hospital, and local hospital). The Charlson comorbidity index (CCI, scores of 0, 1, 2, 3, ≥ 4) is the most widely used comorbidity index^40,41^. Other comorbidities included pneumonia, bronchiectasis, pneumoconiosis, pulmonary alveolar pneumonopathy, chronic obstructive pulmonary disease, asthma, hypertension, stroke, coronary artery disease, diabetes mellitus, chronic kidney disease, osteoporosis, depression, anxiety, hyperlipidemia, smoking-related diseases, and dementia (Table S1).

### Major outcome

All of the study participants were followed from the index date until the onset of lung cancer, death, withdrawal from the NHI program, or the end of 2015. Patients with dementia were identified by the ICD-9-CM codes of lung cancer (ICD-9-CM code: 162).

### Statistical analysis

All analyses were performed using the SPSS software version 22.0 for Windows (IBM Corp., Armonk, NY). χ^2^ and *t* tests were used to evaluate the distribution of the categorical and continuous variables between the patients who did and did not use AChEI. The Fisher exact test for categorical variables was used to statistically examine the differences between the two cohorts. The Multivariate Cox regression model and regression analysis were used so as to determine the risk of lung cancer, and the results are presented as a hazard ratio (HR) with a 95% confidence interval (CI). Differences in the risk of lung cancer between the two groups were estimated using the Kaplan–Meier method with the log-rank test. A 2-tailed p value < 0.05 was considered to be statistically significant.

## Conclusion

In conclusion, this study has shown that the antidepressant users might have a nearly 1.5-fold risk of lung cancer than the non-users in the patients with age ≥ 65. It was also noteworthy for the patients who took a longer duration of rivastigmine and galantamine usage. Therefore, clinicians should be cautious in balancing the benefit and risk of the usage of AChEI in patients with AD.

## Supplementary Information

Supplementary Table S1.

Supplementary Table S2.

Supplementary Table S3.

Supplementary Figure S1.

Supplementary Legends.
