# Supplementary material for: Association between long-term usage of acetylcholinesterase inhibitors and lung cancer in the elderly: a nationwide cohort study
Source: Sci Rep. 2022 Mar 3;12:3531. doi: 10.1038/s41598-022-06377-3 (PMC8894396; doi:10.1038/s41598-022-06377-3)
Supplement: Supplementary file 1 — Supplementary Table S1. [file 41598_2022_6377_MOESM1_ESM.docx]

| **Table S1. Abbreviation, ICD-9-CM, and definition** |  |
| --- | --- |
|  | **ICD-9-CM** |
| **Events: Lung cancers** | 161, with catastrophic illness |
| **Comorbidities** |  |
| **Pneumonia** | 480-486 |
| **Bronchiectasis** | 494 |
| **Pneumoconiosis** | 500-505 |
| **Pulmonary alveolar pneumonopathy** | 516 |
| **Chronic obstructive pulmonary disease** | 491-492, 496 |
| **Asthma** | 493 |
| **Hypertension** | 401-405 |
| **Stroke** | 430-438 |
| **Coronary artery disease** | 410-414 |
| **Diabetes mellitus** | 250 |
| **Chronic kidney disease** | 585 |
| **Osteoporosis** | 733.0 |
| **Depression** | 296.2-296.3, 300.4, 309.1, 309.28, 311 |
| **Anxiety** | 300 |
| **Hyperlipidemia** | 272 |
| **Smoking-related diseases** | 305.1, 518.1, 518.3-518.4, 794.2, V15.82 |
| **Dementia** | 290, 294.1, 331.0 |
| **Charlson comorbidity index revised** | CCI removed lung cancers, COPD, HTN, stroke, CAD, DM, CKD, and dementia |

**ICD-9-CM=International Classification of Diseases, Clinical Modifications**
