# Supplementary material for: Association between long-term usage of acetylcholinesterase inhibitors and lung cancer in the elderly: a nationwide cohort study
Source: Sci Rep. 2022 Mar 3;12:3531. doi: 10.1038/s41598-022-06377-3 (PMC8894396; doi:10.1038/s41598-022-06377-3)
Supplement: Supplementary file 2 — Supplementary Table S2. [file 41598_2022_6377_MOESM2_ESM.docx]

| **Table S2. Characteristics of study at the endpoint** | | | | | |
| --- | --- | --- | --- | --- | --- |
| **AChEI** | **With** | | **Without** | | ***P*** |
| **Variables** | **n** | **%** | **n** | **%** |  |
| **Total** | 116,106 | 25.00 | 348,318 | 75.00 |  |
| **Gender** |  |  |  |  | 0.999 |
| **Lung cancers** |  |  |  |  | 0.770 |
| Without | 111,393 | 95.94 | 334,247 | 95.96 |  |
| With | 4,713 | 4.06 | 14,071 | 4.04 |  |
| Male | 59,052 | 50.86 | 177,156 | 50.86 |  |
| Female | 57,054 | 49.14 | 171,162 | 49.14 |  |
| **Age (yrs)** | 68.21 ± 19.56 | | 70.12 ± 20.45 | | <0.001 |
| **Age groups (yrs)** |  |  |  |  |  |
| 50-64 | 57,598 | 49.61 | 170,240 | 48.87 |  |
| ≧65 | 58,508 | 50.39 | 178,078 | 51.13 |  |
| **Insured premium (NT$)** |  |  |  |  | 0.020 |
| <18,000 | 92,246 | 79.45 | 278,012 | 79.82 |  |
| 18,000-34,999 | 13,459 | 11.59 | 39,454 | 11.33 |  |
| ≧35,000 | 10,401 | 8.96 | 30,852 | 8.86 |  |
| **Marital status** |  |  |  |  | <0.001 |
| Without | 50,985 | 43.91 | 158,636 | 45.54 |  |
| With | 65,121 | 56.09 | 189,682 | 54.46 |  |
| **Education levels (yrs)** |  |  |  |  | <0.001 |
| <12 | 60,986 | 52.53 | 191,336 | 54.93 |  |
| ≧12 | 55,120 | 47.47 | 156,982 | 45.07 |  |
| **Pneumonia** | 8,901 | 7.67 | 14,982 | 4.30 | <0.001 |
| **Bronchiectasis** | 9,201 | 7.92 | 19,898 | 5.71 | <0.001 |
| **Pneumoconiosis** | 8,975 | 7.73 | 16,882 | 4.85 | <0.001 |
| **PAP** | 1,642 | 1.41 | 1,875 | 0.54 | <0.001 |
| **COPD** | 11,023 | 9.49 | 22,780 | 6.54 | <0.001 |
| **Asthma** | 5,652 | 4.87 | 16,702 | 4.80 | 0.315 |
| **Hypertension** | 22,986 | 19.80 | 65,232 | 18.73 | <0.001 |
| **Stroke** | 15,121 | 13.02 | 35,112 | 10.08 | <0.001 |
| **Coronary artery disease** | 18,701 | 16.11 | 35,982 | 10.33 | <0.001 |
| **Diabetes mellitus** | 24,982 | 21.52 | 70,154 | 20.14 | <0.001 |
| **Chronic Kidney Disease** | 16,701 | 14.38 | 46,444 | 13.33 | <0.001 |
| **Osteoporosis** | 3,355 | 2.89 | 6,201 | 1.78 | <0.001 |
| **Depression** | 6,825 | 5.88 | 4,142 | 1.19 | <0.001 |
| **Anxiety** | 5,722 | 4.93 | 3,012 | 0.86 | <0.001 |
| **Hyperlipidemia** | 5,176 | 4.46 | 12,045 | 3.46 | <0.001 |
| **Smoking-related diseases** | 4,923 | 4.24 | 13,551 | 3.89 | <0.001 |
| **Dementia** | 29,886 | 25.74 | 68,124 | 19.56 | <0.001 |
| **CCI_R** | 1.03 ± 1.88 | | 0.86 ± 1.77 | | <0.001 |
| **Location** |  |  |  |  | <0.001 |
| Northern Taiwan | 43,298 | 37.29 | 130,697 | 37.52 |  |
| Middle Taiwan | 28,441 | 24.50 | 90,884 | 26.09 |  |
| Southern Taiwan | 32,095 | 27.64 | 98,898 | 28.39 |  |
| Eastern Taiwan | 10,645 | 9.17 | 25,201 | 7.24 |  |
| Outlets islands | 1,627 | 1.40 | 2,638 | 0.76 |  |
| **Urbanization level** |  |  |  |  | <0.001 |
| 1 (The highest) | 40,101 | 34.54 | 117,796 | 33.82 |  |
| 2 | 45,164 | 38.90 | 139,264 | 39.98 |  |
| 3 | 10,186 | 8.77 | 34,121 | 9.80 |  |
| 4 (The lowest) | 20,655 | 17.79 | 57,137 | 16.40 |  |
| **Level of care** |  |  |  |  | <0.001 |
| Hospital center | 44,602 | 38.41 | 111,624 | 32.05 |  |
| Regional hospital | 42,201 | 36.35 | 129,712 | 37.24 |  |
| Local hospital | 29,303 | 25.24 | 106,982 | 30.71 |  |
| ***P:* Chi-square / Fisher exact test on category variables and t-test on continue variables**  **AChEI=Acetylcholinesterase Inhibitors; CCI_R=Charlson Comorbidity Index, dementia removed; COPD=chronic obstructive pulmonary disease; NT$=New Taiwan Dollars; PAP=Pulmonary alveolar pneumonopathy** | | | | | |
