# Supplementary material for: Association between long-term usage of acetylcholinesterase inhibitors and lung cancer in the elderly: a nationwide cohort study
Source: Sci Rep. 2022 Mar 3;12:3531. doi: 10.1038/s41598-022-06377-3 (PMC8894396; doi:10.1038/s41598-022-06377-3)
Supplement: Supplementary file 3 — Supplementary Table S3. [file 41598_2022_6377_MOESM3_ESM.docx]

| **Table S3. Factors of lung cancers stratified by variables listed in the table by using Cox regression and Fine & Gray's competing risk model** | | | | |
| --- | --- | --- | --- | --- |
| **AChEI** |  | | | |
| **Stratified** | **Adjusted HR** | **95% CI** | **95% CI** | ***P*** |
| **Total** | 1.198 | 0.765 | 1.774 | 0.167 |
| **Gender** |  |  |  |  |
| Male | 1.378 | 0.880 | 2.040 | 0.295 |
| Female | 1.013 | 0.647 | 1.500 | 0.165 |
| **Age groups (yrs)** |  |  |  |  |
| 50-64 | 1.060 | 0.677 | 1.570 | 0.189 |
| ≧65 | 1.329 | 0.849 | 1.968 | 0.244 |
| **Insured premium (NT$)** |  |  |  |  |
| <18,000 | 1.079 | 0.689 | 1.597 | 0.498 |
| 18,000-34,999 | 1.403 | 0.896 | 2.077 | 0.385 |
| ≧35,000 | 1.419 | 0.906 | 2.101 | 0.301 |
| **Marital status** |  |  |  |  |
| Without | 1.082 | 0.691 | 1.602 | 0.298 |
| With | 1.270 | 0.811 | 1.881 | 0.131 |
| **Education levels (yrs)** |  |  |  |  |
| <12 | 1.169 | 0.747 | 1.732 | 0.225 |
| ≧12 | 1.185 | 0.757 | 1.755 | 0.187 |
| **Pneumonia** |  |  |  |  |
| Without | 0.989 | 0.631 | 1.464 | 0.265 |
| With | 1.653 | 1.055 | 2.448 | <0.001 |
| **Bronchiectasis** |  |  |  |  |
| Without | 1.153 | 0.737 | 1.708 | 0.201 |
| With | 1.380 | 0.881 | 2.044 | 0.198 |
| **Pneumoconiosis** |  |  |  |  |
| Without | 1.005 | 0.642 | 1.489 | 0.642 |
| With | 1.713 | 1.094 | 2.537 | <0.001 |
| **Pulmonary alveolar pneumonopathy** |  |  |  |  |
| Without | 1.198 | 0.765 | 1.773 | 0.265 |
| With | 1.402 | 0.895 | 2.077 | 0.301 |
| **COPD** |  |  |  |  |
| Without | 1.212 | 0.774 | 1.795 | 0.279 |
| With | 1.441 | 0.920 | 2.134 | 0.284 |
| **Asthma** |  |  |  |  |
| Without | 1.198 | 0.765 | 1.774 | 0.138 |
| With | 1.308 | 0.835 | 1.937 | 0.198 |
| **HTN** |  |  |  |  |
| Without | 1.181 | 0.754 | 1.749 | 0.178 |
| With | 1.463 | 0.934 | 2.166 | 0.089 |
| **Stroke** |  |  |  |  |
| Without | 1.194 | 0.762 | 1.768 | 0.231 |
| With | 1.247 | 0.796 | 1.847 | 0.259 |
| **CAD** |  |  |  |  |
| Without | 1.229 | 0.785 | 1.820 | 0.386 |
| With | 1.064 | 0.679 | 1.575 | 0.402 |
| **DM** |  |  |  |  |
| Without | 1.186 | 0.757 | 1.756 | 0.201 |
| With | 1.414 | 0.903 | 2.094 | 0.245 |
| **CKD** |  |  |  |  |
| Without | 1.116 | 0.713 | 1.652 | 0.419 |
| With | 1.637 | 1.046 | 2.425 | 0.001 |
| **Osteoporosis** |  |  |  |  |
| Without | 1.208 | 0.772 | 1.789 | 0.501 |
| With | 1.298 | 0.829 | 1.922 | 0.459 |
| **Depression** |  |  |  |  |
| Without | 1.214 | 0.775 | 1.798 | 0.352 |
| With | 1.222 | 0.780 | 1.809 | 0.398 |
| **Anxiety** |  |  |  |  |
| Without | 1.195 | 0.763 | 1.770 | 0.282 |
| With | 1.217 | 0.777 | 1.802 | 0.115 |
| **Hyperlipidemia** |  |  |  |  |
| Without | 1.182 | 0.755 | 1.750 | 0.245 |
| With | 1.452 | 0.927 | 2.151 | 0.184 |
| **Smoking-related diseases** |  |  |  |  |
| Without | 1.201 | 0.767 | 1.778 | 0.313 |
| With | 1.189 | 0.760 | 1.761 | 0.288 |
| **Dementia** |  |  |  |  |
| Without | 1.192 | 0.761 | 1.765 | 0.187 |
| With | 1.386 | 0.885 | 2.052 | 0.245 |
| **Urbanization level** |  |  |  |  |
| 1 (The highest) | 1.338 | 0.854 | 1.981 | 0.398 |
| 2 | 1.215 | 0.776 | 1.799 | 0.348 |
| 3 | 1.019 | 0.651 | 1.509 | 0.305 |
| 4 (The lowest) | 1.003 | 0.641 | 1.486 | 0.298 |
| **Level of care** |  |  |  |  |
| Hospital center | 1.143 | 0.730 | 1.692 | 0.603 |
| Regional hospital | 1.138 | 0.726 | 1.684 | 0.597 |
| Local hospital | 1.090 | 0.696 | 1.614 | 0.548 |
| **PYs = Person-years; Adjusted HR = Adjusted Hazard ratio: Adjusted for the variables listed in Table 3.; CI = confidence interval** | | | | |
