# Supplementary figures and images for: Association between long-term usage of acetylcholinesterase inhibitors and lung cancer in the elderly: a nationwide cohort study
Source: Sci Rep. 2022 Mar 3;12:3531. doi: 10.1038/s41598-022-06377-3 (PMC8894396; doi:10.1038/s41598-022-06377-3)

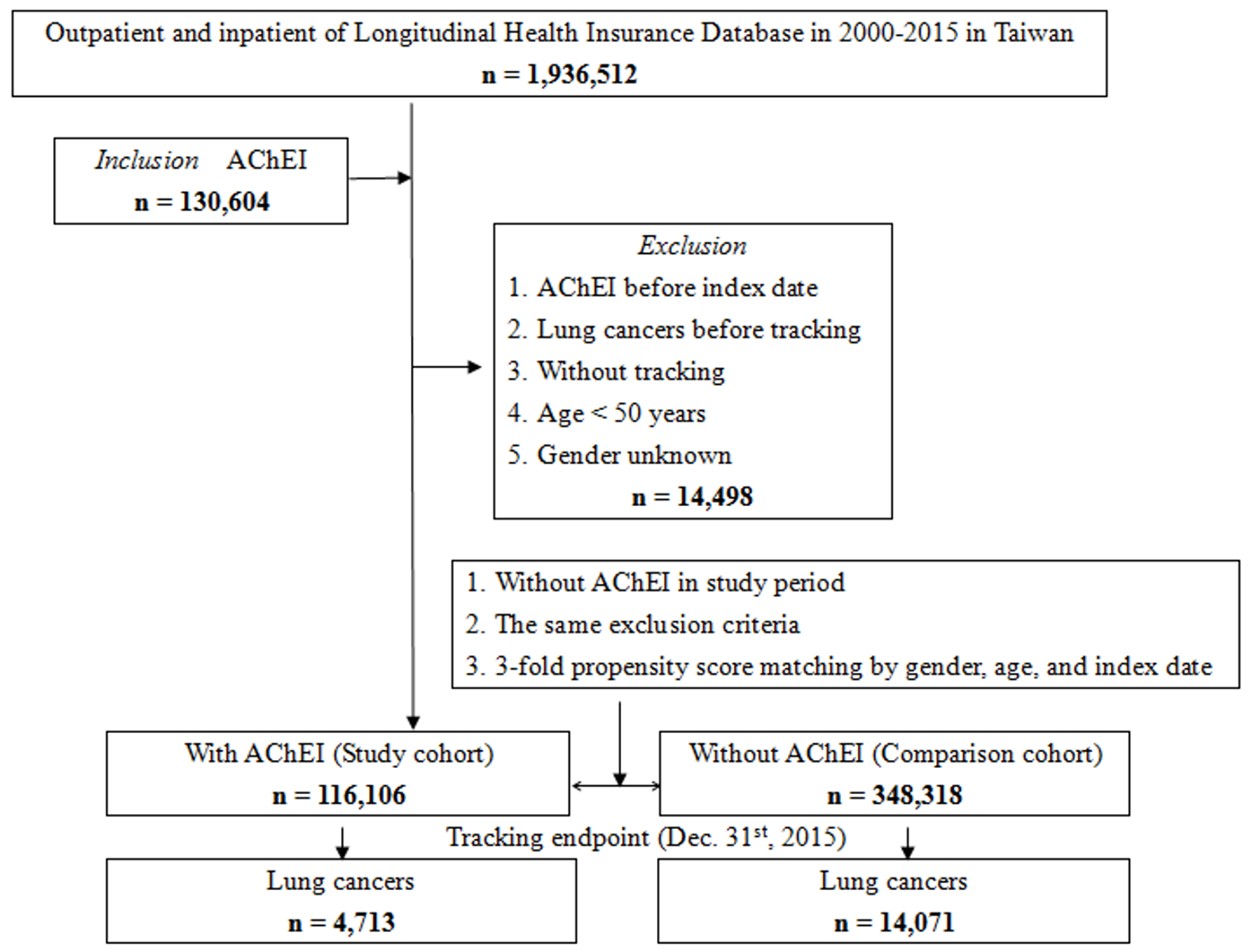

Supplement: Supplementary file 4 — Supplementary Figure S1. [file 41598_2022_6377_MOESM4_ESM.tiff]
